# Glycemic status and risks of thromboembolism and major bleeding in patients with atrial fibrillation

**DOI:** 10.1186/s12933-020-01005-8

**Published:** 2020-03-10

**Authors:** Yi-Hsin Chan, Chi Chuang, Cze-Ci Chan, Hsin-Fu Lee, Ya-Chi Huang, Yu-Tung Huang, Shang-Hung Chang, Chun-Li Wang, Tze-Fan Chao, Chi-Tai Kuo, Yung-Hsin Yeh, Shih-Ann Chen

**Affiliations:** 1 The Cardiovascular Department, Chang Gung Memorial Hospital, Linkou, 33305 Taoyuan Taiwan; 2 College of Medicine, Chang Gung University, Taoyuan, 33302 Taiwan; 3 Microscopy Core Laboratory, Chang Gung Memorial Hospital, Linkou, 33305 Taoyuan Taiwan; 4 Graduate Institute of Clinical Medical Sciences, College of Medicine, Chang Gung University, Taoyuan, Taiwan; 5 Center for Big Data Analytics and Statistics, Chang Gung Memorial Hospital, Taoyuan, Taiwan; 6 Division of Cardiology, Department of Medicine, Taipei Veterans General Hospital, No. 201, Sec. 2, Shih-Pai Road, Taipei, Taiwan; 7 Institute of Clinical Medicine and Cardiovascular Research Center, National Yang-Ming University, Taipei, Taiwan

**Keywords:** Atrial fibrillation, HbA1c, Ischemic stroke, Major bleeding, Direct oral anticoagulants, Warfarin

## Abstract

**Background:**

Studies specifically examining the association between glycated hemoglobin A1c (HbA1c) levels and ischemic stroke/systemic thromboembolism (IS/SE) risk in atrial fibrillation (AF) patients are limited. Here, we investigated the association between HbA1c levels and the risk of IS/SE, as well as major bleeding, among AF patients with or without oral anticoagulants (OACs). We also compared the effectiveness and safety of warfarin and direct oral anticoagulants (DOACs) in different HbA1c categories.

**Methods:**

We utilized medical data from a multi-center healthcare provider in Taiwan, which included 34,036 AF patients with serum HbA1c data available within 3 months after AF being diagnosed. Patients were divided into seven study groups according to their HbA1c levels: < 5.4%, 5.4%–5.6%, 5.7%–5.9%, 6.0%–6.4%, 6.5%–6.9%, 7.0%–7.9%, and ≥ 8.0%. The risks of IS/SE and major bleeding were compared among the groups after adjusting for baseline stroke and bleeding risk factors.

**Results:**

Compared with the patients with HbA1c level < 5.4%, IS/SE risk significantly increased at HbA1c levels higher than 6.5% [adjusted hazard ratio (HR): 1.20, 95% confidence interval (CI): 1.00–1.43 for HbA1c level 6.5%–6.9%; 1.32, (95% CI 1.11–1.57) for HbA1c level 7.0%–7.9%; and 1.48 (95% CI 1.25–1.76) for HbA1c level ≥ 8.0%]. These results were generally consistent in AF patients without OACs (n = 24,931). However, among 9105 patients receiving OACs, IS/SE risk was not higher for patients having higher HbA1c levels. The risk of major bleeding was comparable across all HbA1c categories. Compared with warfarin, DOACs were associated with lower risks of IS/SE (adjusted HR: 0.61, 95% CI 0.49–0.75) and major bleeding (adjusted HR: 0.30, 95% CI 0.21–0.42) without interactions across different HbA1c categories (all *P* interactions > 0.05).

**Conclusion:**

For AF patients, IS/SE risk significantly increased once HbA1c levels exceeded 6.5%, and OACs may attenuate these associations. Compared with warfarin, DOACs were more effective and safer across broad HbA1c categories. Therefore, in addition to prescribing DOACs when indicated, more aggressive glycemic control to achieve an HbA1c level < 6.5% may be considered for eligible AF patients and should be tested in further prospective studies.

## Background

Diabetes mellitus (DM), insulin resistance, and obesity are among the major risk factors for atrial fibrillation (AF) [1, 2]. DM is also a crucial risk factor in many widely used stroke prediction schemes (e.g., CHADS_2_ and CHA_2_DS_2_-VASc scores) for patients with AF [3, 4]. Glycated hemoglobin A1c (HbA1c) is an indicator of long-term glycemic control, and elevated HbA1c is a reliable marker reflecting chronic hyperglycemia [5, 6]. Previous studies indicated a significant dose–response association between HbA1c levels and the risk of stroke in DM patients without AF [7–9]. However, studies that specifically examine the association between HbA1c levels and the risk of thromboembolism in AF patients have been limited and showed conflicting data [10–12]. Therefore, it remains unclear whether poor glycemic control, represented by high HbA1c, is significantly associated with an increased risk of ischemic stroke/systemic thromboembolism (IS/SE) among AF patients. In addition, although DM itself is not currently considered as an independent risk factor of bleeding in the bleeding scheme [13], recent studies indicated that it could be independently associated with an increased risk of major bleeding in AF patients [14, 15].

In this study, we investigated the association between HbA1c levels and the risks of IS/SE as well as major bleeding in around 34,000 AF patients with or without oral anticoagulants (OACs) using a large multicenter-based electronic medical registry database. Besides, we also compared the effectiveness and safety of warfarin and direct oral anticoagulants (DOACs) among AF patients in different HbA1c categories.

## Methods

This study was approved by the Institutional Review Board of the Chang Gung Medical Foundation and based on the data from Chang Gung Research Database provided by Chang Gung Memorial Hospital. The interpretation and conclusions presented here do not represent the position of Chang Gung Memorial Hospital.

### Database

All patients’ data until 2019 were collected from the Chang Gung Memorial Hospital (CGMH) Medical System, which is the largest healthcare provider in Taiwan. The CGMH Medical System consists of four tertiary care medical centers and three major teaching hospitals, with total hospital beds of up to 10,000 and around 280,000 patients being admitted per year [16]. In 2015, there were approximately 500,000 emergency department visits and 8,600,000 outpatient department visits to the CGMH, accounting for 10% of the Taiwanese medical service annually [16, 17]. The advantage of the CGMH medical database is that each patient’s detailed chart record, diagnosis, imaging, and laboratory data are all available [18]. The identification number and personal information of each patient were encrypted and de-identified by using a consistent encrypting procedure; therefore, informed consent was waived for this study.

### Study population

**Fig. 1:**
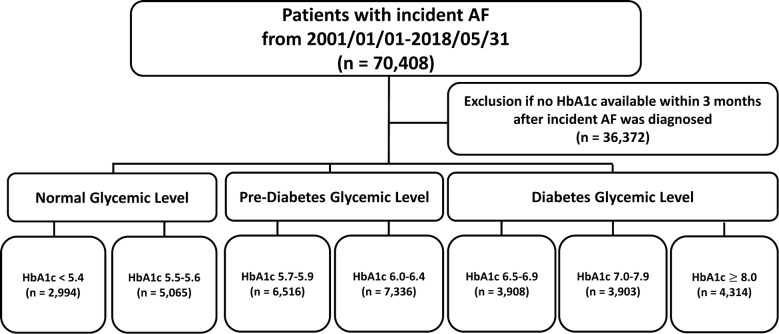
The flowchart of the enrollment of study patients. From June 1, 2001, to May 31, 2018, 2944, 5065, 6516, 7336, 3908, 3903, and 4314 AF patients with HbA1c levels of < 5.4%, 5.4%–5.6%, 5.7%–5.9%, 6.0%–6.4%, 6.5%–6.9%, 7.0%–7.9%, and $\ge$ 8.0%, respectively, were enrolled in the present study. AF = atrial fibrillation; HbA1c = glycated hemoglobin A1c

We retrospectively studied 70,408 patients over 20 years old in whom new-onset AF was diagnosed between January 1, 2001, and May 31, 2018, from the CGMH medical database. There were 34,036 patients with serum HbA1c data within 3 months after the diagnosis of AF. The index date was defined as the date of baseline HbA1c in the CGMH Patient Register during the inclusion period. First, we categorized our study population into no-DM (HbA1c < 5.7%; n = 8059), pre-DM (5.7%–6.4%; n = 13,852), and DM ($\ge$ 6.5%; n = 12,125) subgroups according to the ADA guidelines [19]. Subsequently, we further divided our patients into two [< 5.4% (n = 2994) and 5.5%–5.6% (n = 5065)], two [5.7%–5.9% (n = 6526) and 6.0%–6.4% (7336)], and three [6.5%–6.9% (n = 3908), 7.0%–7.9% (n = 3903), and $\ge$ 8.0% (n = 4314)] subgroups among the no-DM, pre-DM, and DM subgroups. The cut-off values used within the pre-DM (< 5.4% and 5.5%–5.6%) and DM (6.5%–6.9%, 7.0%–7.9%, and $\ge$ 8.0%) subgroups were chosen to result in similar patient numbers between these subgroups. We set HbA1c < 5.4% as the reference group in the present study because an HbA1c level of 5.4% corresponds to fasting plasma glucose of 100 mg/dl [19]. Patients were finally divided into seven study groups according to their baseline HbA1c levels: HbA1c of < 5.4% (n = 2994), 5.4%–5.6% (n = 5065), 5.7%–5.9% (n = 6516), 6.0%–6.4% (n = 7336), 6.5%–6.9% (n = 3908), 7.0%–7.9% (n = 3903), and $\ge$ 8.0% (n = 4314). A flow chart of the study is presented in Fig. 1.

### Follow-up and outcome

The clinical outcomes of the present study were the first occurrences of hospitalized IS/SE and major bleeding. The diagnosis of IS/SE should be confirmed by a corresponding chart record plus medical imaging including brain computed tomography/magnetic resonance imaging. The major bleeding events were defined as hospitalization due to symptomatic bleeding in a critical organ including brain, gastrointestinal tract, or other critical sites, bleeding causing a decrease in hemoglobin level of **≥**2 g/dL, bleeding leading to blood transfusion of **≥**2 units, or fatal bleeding. All study outcomes were defined based on first discharge diagnosis to avoid misclassifications. Patients were followed up from the date of first recorded HbA1c until the first occurrence of any study outcome, mortality, or end date of the study (May 31, 2018).

### Covariates

Baseline covariates were obtained from any claim records with the diagnoses, procedures, or medication codes before the index date. Baseline prescription medicine was confined to medications taken at least once within 3 months before the index date. Bleeding history was confined to events within 6 months preceding the index date. The CHA_2_DS_2_-VASc score (congestive heart failure, hypertension, age 75 years or older for 2 points, DM, previous stroke or transient ischemic attack for 2 points, vascular disease, age 65 to 74 years, and female gender) was used to predict the risk of IS/SE in AF patients [20, 21]. The HAS-BLED score [hypertension, abnormal renal or liver function, stroke, bleeding history, labile international normal ratio (INR), age 65 years or older, and antiplatelet drug or alcohol use] was used to predict the risk of bleeding in AF patients treated with OACs [13].

### Statistical analysis

Data are presented as mean and standard deviation for continuous variables and as proportions for categorical variables. ANOVA was used to analyze the differences for continuous variables. χ^2^ test was used to analyze the differences for nominal variables. Crude incidence rates were computed as the total number of study outcomes during the follow-up time divided by person-years at risk. Multivariate Cox proportional hazards regression was used to compare the risk of events in AF patients across different HbA1c categories. Statistical significance was defined as a *P* value of < 0.05. All analyses were conducted using SAS 9.2 (SAS Institute Inc., Cary, NC, USA).

## Results

### Baseline characteristics

The clinical characteristics of AF patients in different HbA1c categories are shown in Table 1. In general, patients with a higher baseline HbA1c level had a higher CHA_2_DS_2_-VASc score, a higher prevalence of dyslipidemia, heavier body weight, and a lower estimated glomerular filtration rate (eGFR). In groups with higher HbA1c levels, a higher percentage received insulin or oral hypoglycemic agents (OHAs), angiotensin-converting-enzyme inhibitors (ACEIs)/angiotensin II receptor antagonists (ARBs), and statins (*P* < 0.0001). Approximately 60% of patients had baseline echocardiography data in each HbA1c group. In general, patients with a higher baseline HbA1c level had a lower left ventricular ejection fraction value (*P* < 0.0001).

**Table 1:** Baseline characteristics of AF patients in different HbA1c categories

	Glycemic status represented by HbA1c							
	HbA1c < 5.4 (n = 2994)	HbA1c 5.4–5.6 (n = 5065)	HbA1c 5.7–5.9 (n = 6516)	HbA1c 6.0–6.4 (n = 7336)	HbA1c 6.5–6.9 (n = 3908)	HbA1c 7.0–7.9 (n = 3903)	HbA1c ≥ 8.0 (n = 4314)	*P* value (ANOVA)
Age, yrs	69.1 (14.3)	69.1 (13.5)	69.7 (12.8)	70.6 (11.8)	71.2 (11.1)	70.6 (11.0)	68.9 (11.2)	< 0.0001
Body weight, kg	61.0 (13.8)	63.5 (13.4)	64.5 (13.7)	66.1 (14.0)	66.9 (14.4)	67.2 (14.2)	67.1 (13.8)	< 0.0001
Stroke comorbidity								
CHA_2_DS_2_-VASc score	2.9 (1.9)	2.8 (1.8)	2.9 (1.8)	3.1 (1.8)	3.5 (1.9)	3.6 (1.8)	3.5 (1.8)	< 0.0001
Congestive heart failure	733 (24.5)	1064 (21.0)	1350 (20.7)	1626 (22.2)	989 (25.3)	967 (24.8)	1103 (25.6)	< 0.0001
Hypertension	1532 (51.2)	2591 (51.2)	3475 (53.3)	4229 (57.7)	2427 (62.1)	2424 (62.1)	2486 (57.6)	< 0.0001
Age ≥ 75 yrs	1198 (40.0)	1898 (37.5)	2485 (38.1)	2899 (39.5)	1535 (39.3)	1460 (37.4)	1418 (32.9)	< 0.0001
Diabetes Mellitus	396 (13.2)	625 (12.3)	939 (14.4)	1873 (25.5)	1907 (48.8)	2477 (63.5)	2733 (63.4)	< 0.0001
Previous stroke or TIA	658 (22.0)	1080 (21.3)	1302 (20.0)	1417 (19.3)	765 (19.6)	780 (20.0)	863 (20.0)	0.0224
Vascular disease	425 (14.2)	684 (13.5)	875 (13.4)	1069 (14.6)	698 (17.9)	755 (19.3)	820 (19.0)	< 0.0001
Age 65–74 yrs	665 (22.2)	1231 (24.3)	1624 (24.9)	1961 (26.7)	1116 (28.6)	1205 (30.9)	1209 (28.0)	< 0.0001
Female	1263 (42.2)	2081 (41.1)	2823 (43.3)	3192 (43.5)	1801 (46.1)	1809 (46.4)	2001 (46.4)	< 0.0001
Bleeding comorbidity								
HAS-BLED score	2.8 (1.4)	2.6 (1.3)	2.6 (1.3)	2.7 (1.2)	2.8 (1.2)	2.8 (1.3)	2.7 (1.3)	< 0.0001
Age ≥ 65 yrs	1965 (65.6)	3292 (65.0)	4347 (66.7)	5094 (69.4)	2812 (72.0)	2803 (71.8)	2781 (64.5)	< 0.0001
Chronic liver disease	640 (21.4)	798 (15.8)	995 (15.3)	1079 (14.7)	610 (15.6)	625 (16.0)	655 (15.2)	< 0.0001
Chronic kidney disease	685 (22.9)	925 (18.3)	1030 (15.8)	1178 (16.1)	698 (17.9)	774 (19.8)	761 (17.6)	< 0.0001
Previous History of bleeding	215 (7.2)	287 (5.7)	362 (5.6)	386 (5.3)	243 (6.2)	212 (5.4)	226 (5.2)	0.0037
Previous stroke or TIA	658 (22.0)	1080 (21.3)	1302 (20.0)	1417 (19.3)	765 (19.6)	780 (20.0)	863 (20.0)	0.0224
Use of NSAIDs	1758 (58.7)	2845 (56.2)	3698 (56.8)	4120 (56.2)	2163 (55.4)	2131 (54.6)	2301 (53.3)	< 0.0001
Other comorbidity								
Hyperlipidemia	714 (23.9)	1293 (25.5)	1943 (29.8)	2389 (32.6)	1396 (35.7)	1515 (38.8)	1523 (35.3)	< 0.0001
Gout	417 (13.9)	693 (13.7)	837 (12.9)	1035 (14.1)	521 (13.3)	492 (12.6)	519 (12.0)	0.0232
Malignancy	321 (10.7)	465 (9.2)	659 (10.1)	683 (9.3)	406 (10.4)	435 (11.2)	399 (9.3)	0.0056
Laboratory data								
Hemoglobin, g/dl	12.1 (2.4)	12.7 (2.3)	12.9 (2.2)	12.9 (2.2)	12.6 (2.3)	12.5 (2.3)	12.8 (2.3)	< 0.0001
Platelet, ×1000/Ul	187.1 (76.6)	191.4 (73.2)	198.3 (74.0)	201.9 (74.3)	206.4 (78.4)	208.4 (80.9)	209.2 (84.2)	< 0.0001
eGFR, ml/min/1.73 m^2^	69.3 (41.0)	70.6 (34.8)	72.0 (34.0)	70.0 (31.8)	67.2 (36.5)	65.0 (34.8)	64.1 (36.6)	< 0.0001
HbA1c, %	5.2 (0.50)	5.6 (0.3)	5.8 (0.4)	6.2 (0.5)	6.8 (0.7)	7.5 (0.9)	9.5 (1.9)	< 0.0001
Baseline echocardiography								
Number of patients with data of echocardiography	1832 (61.2)	3172 (62.2)	4207 (64.6)	4657 (63.5)	2514 (64.3)	2509 (64.3)	2562 (59.4)	
LVEF, %	62.6 (13.6)	62.7 (14.1)	62.3 (13.8)	61.9 (14.6)	61.3 (14.4)	61.1 (14.4)	60.8 (14.6)	< 0.0001
Baseline medications								
Use of Insulin	146 (4.9)	253 (5.0)	337 (5.2)	633 (8.6)	670 (17.1)	1080 (27.7)	1786 (41.4)	< 0.0001
Use of OHAs, not including SGLT2i and GLP1 agonist	208 (6.9)	308 (6.1)	554 (8.5)	1321 (18.0)	1442 (36.9)	1930 (49.4)	2103 (48.7)	< 0.0001
Use of SGLT2i	15 (0.5)	45 (0.9)	68 (1.0)	187 (2.6)	212 (5.4)	365 (9.4)	526 (12.2)	< 0.0001
Use of GLP1 agonist	0 (0)	0 (0)	0 (0)	0 (0)	1 (0.0)	1 (0.0)	3 (0.1)	0.0028
Use of warfarin	459 (15.3)	796 (15.7)	1051 (16.1)	1082 (14.8)	579 (14.8)	563 (14.4)	609 (14.1)	0.0498
Use of DOACs	264 (8.8)	541 (10.7)	824 (12.7)	1030 (14.0)	513 (13.1)	438 (11.2)	356 (8.3)	< 0.0001
Use of NSAIDs	410 (13.7)	588 (11.6)	739 (11.3)	947 (12.9)	508 (13.0)	494 (12.7)	601 (13.9)	< 0.0001
Use of ACEI/ARB	1209 (40.4)	2167 (42.8)	2855 (43.8)	3559 (48.5)	1966 (50.3)	2006 (51.4)	2048 (47.5)	< 0.0001
Use of loop diuretics	842 (28.1)	1257 (24.8)	1591 (24.4)	1993 (27.2)	1262 (32.3)	1265 (32.4)	1493 (34.6)	< 0.0001
Use of amiodarone	577 (19.3)	983 (19.4)	1105 (17.0)	1279 (17.4)	728 (18.6)	678 (17.4)	661 (15.3)	< 0.0001
Use of dronedarone	47 (1.6)	76 (1.5)	103 (1.6)	89 (1.2)	43 (1.1)	42 (1.1)	18 (0.4)	< 0.0001
Use of beta-blocker	1222 (40.8)	2183 (43.1)	2947 (45.2)	3457 (47.1)	2011 (51.5)	1950 (50.0)	2003 (46.4)	< 0.0001
Use of diltiazem	396 (13.2)	721 (14.2)	1025 (15.7)	1145 (15.6)	699 (17.9)	721 (18.5)	756 (17.5)	< 0.0001
Use of verapamil	127 (4.2)	206 (4.1)	258 (4.0)	313 (4.3)	168 (4.3)	184 (4.7)	174 (4.0)	0.6483
Use of digoxin	498 (16.6)	740 (14.6)	1005 (15.4)	1204 (16.4)	698 (17.9)	735 (18.8)	1054 (24.4)	< 0.0001
Use of statin	368 (12.3)	838 (16.5)	1330 (20.4)	1720 (23.5)	1073 (27.5)	1144 (29.3)	1098 (25.5)	< 0.0001

Data are expressed as mean ± standard deviation or as numbers (percentage)

*ACEI* angiotensin-converting-enzyme inhibitor, *AF* atrial fibrillation, *APT* antiplatelet agent, *ARB* angiotensin II receptor antagonists, *CHA*_*2*_*DS*_*2*_*-VASc* congestive heart failure, hypertension, age 75 years or older, diabetes mellitus, previous stroke/transient ischemic attack, vascular disease, age 65 to 74 years, female, *DOACs* direct oral anticoagulants, *eGFR* estimated glomerular filtration rate, *GLP1* glucagon -like peptide-1, *HAS-BLED* hypertension, abnormal renal or liver function, stroke, bleeding history, labile INR, age 65 years or older, and antiplatelet drug or alcohol use, *HbA1c* glycated hemoglobin A1c, *LVEF* left ventricular ejection fraction, *OHAs* oral hypoglycemic agent, *NSAIDs* non-steroidal anti-inflammatory drugs, *SGLT2i* sodium glucose co-transporter-2 inhibitor, *TIA* transient ischemic attack

### Associations between HbA1c and risks of IS/SE and major bleeding

**Fig. 2:**
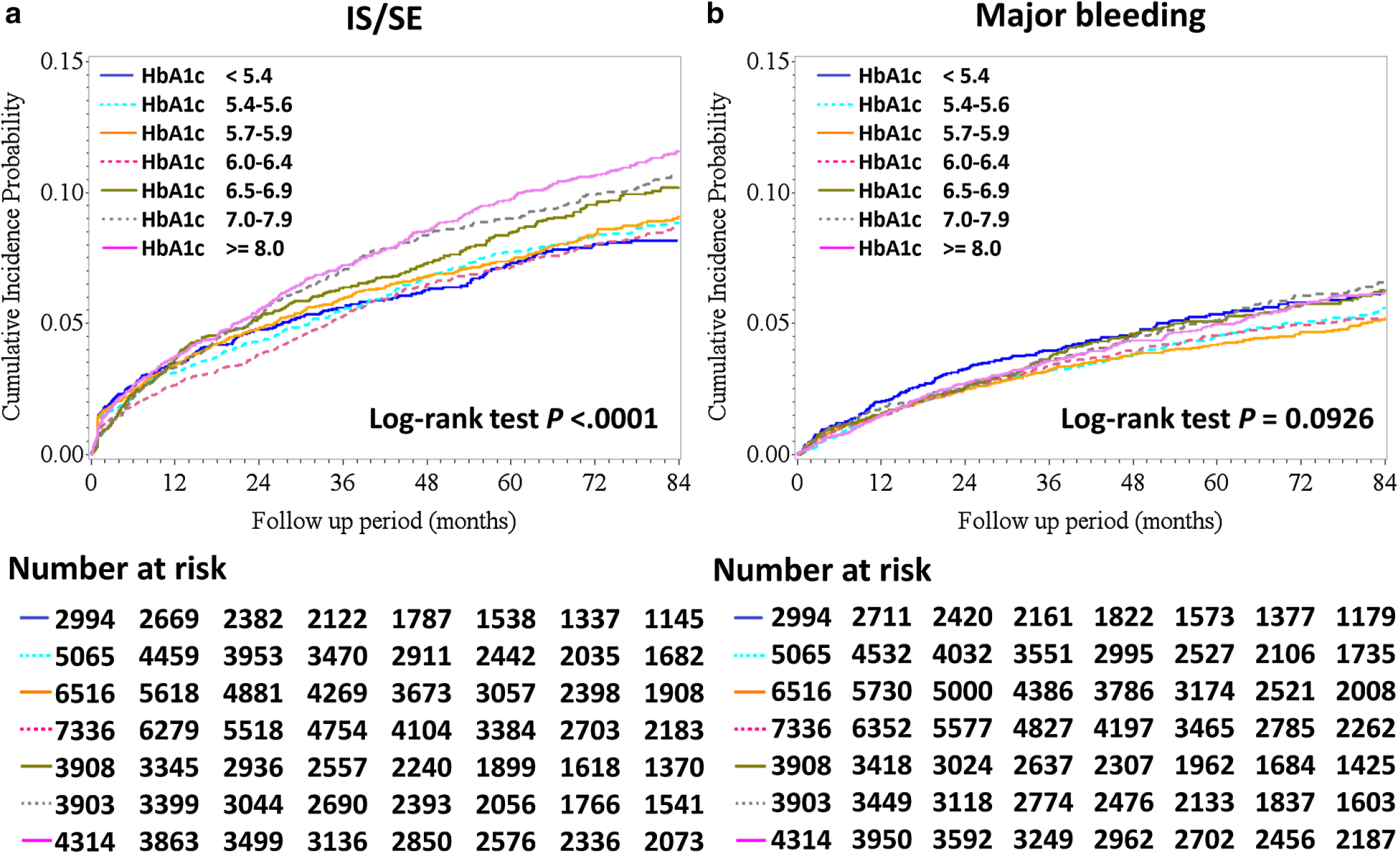
Cumulative incidence curves of IS/SE (**a**) and major bleeding (**b**) among AF patients in different HbA1c categories. Thromboembolic events appeared to be higher in patients with higher HbA1c levels of 6.5%–6.9%, 7.0%–7.9%, and $\ge$ 8.0% than in the other groups (log-rank *P* < 0.0001), while the risk of major bleeding was comparable across all groups (log-rank *P* = 0.0926). AF = atrial fibrillation; HbA1c = glycated hemoglobin A1c; IS/SE = ischemic stroke/systemic embolism

In general, the crude incidence rate of IS/SE of AF patients continuously increased across HbA1c categories, but the crude incidence rates of major bleeding were similar in different HbA1c groups (Table 2). The cumulative incidence curves of IS/SE and major bleeding of different HbA1c groups are shown in Fig. 2. The risk of IS/SE appeared to be higher in patients with a higher HbA1c level (6.5%–6.9%, 7.0%–7.9%, and $\ge$ 8.0%) (log-rank *P* < 0.0001), while the risk of major bleeding did not differ significantly across all groups (log-rank *P* = 0.0926).

**Table 2:** Risks of hospitalized IS/SE and major bleeding among AF patients in different HbA1c categories

	Event rate/100 person-years (95% CI)	Crude		Adjusted	
		HR (95% CI)	*P* value	HR (95% CI)^a^	*P* value
IS/SE					
HbA1c < 5.4	1.20 (1.04–1.36)	1.00 (Reference)	–	1.00 (Reference)	–
HbA1c 5.4–5.6	1.33 (1.20–1.47)	1.06 (0.9–1.26)	0.4926	1.08 (0.92–1.28)	0.3508
HbA1c 5.7–5.9	1.41 (1.29–1.54)	1.1 (0.93–1.29)	0.2576	1.12 (0.96–1.32)	0.1571
HbA1c 6.0–6.4	1.31 (1.20–1.42)	1.02 (0.87–1.2)	0.7701	1.04 (0.88–1.22)	0.6659
HbA1c 6.5–6.9	1.47 (1.31–1.63)	*1.21 (1.01–1.43)*	*0.0341*	*1.2 (1–1.43)*	*0.0450*
HbA1c 7.0–7.9	1.55 (1.40–1.71)	*1.32 (1.12–1.56)*	*0.0011*	*1.32 (1.11–1.57)*	*0.0019*
HbA1c ≥ 8.0	1.53 (1.39–1.67)	*1.39 (1.18–1.63)*	*< 0.0001*	*1.48 (1.25–1.76)*	*< 0.0001*

	Event rate/100 person-years (95% CI)	Crude	Adjusted	HR (95% CI)^b^	*P* value
		HR (95% CI)	*P* value		
Major bleeding					
HbA1c < 5.4	0.84 (0.71–0.97)	1.00 (Reference)	–	1.00 (Reference)	–
HbA1c 5.4–5.6	0.76 (0.65–0.86)	0.86 (0.7–1.06)	0.1554	0.9 (0.73–1.11)	0.3136
HbA1c 5.7–5.9	0.75 (0.66–0.84)	0.84 (0.69–1.02)	0.0770	0.88 (0.72–1.08)	0.2178
HbA1c 6.0–6.4	0.78 (0.69–0.87)	0.88 (0.72–1.06)	0.1770	0.89 (0.73–1.08)	0.2353
HbA1c 6.5–6.9	0.85 (0.73–0.97)	1 (0.81–1.24)	0.9942	0.94 (0.76–1.17)	0.5790
HbA1c 7.0–7.9	0.83 (0.72–0.95)	1.02 (0.83–1.26)	0.8314	0.93 (0.75–1.16)	0.5121
HbA1c ≥ 8.0	0.78 (0.68–0.87)	1.01 (0.83–1.24)	0.9151	0.92 (0.74–1.15)	0.4700

^a^Adjustments for CHA_2_DS_2_-VASc score, use of insulin or OHAs, use of warfarin or DOACs, and eGFR

^b^Adjustments for HAS-BLED score, use of insulin or OHAs, use of warfarin or DOACs, and eGFR

*AF* atrial fibrillation, *CI* confidence interval, *DOACs* direct oral anticoagulants, *eGFR* estimated glomerular filtration rate, *HbA1c* glycated hemoglobin A1c, *HR* hazard ratio, *IS/SE* ischemic stroke or systemic embolism, *OHAs* oral hypoglycemic agents

**Fig. 3:**
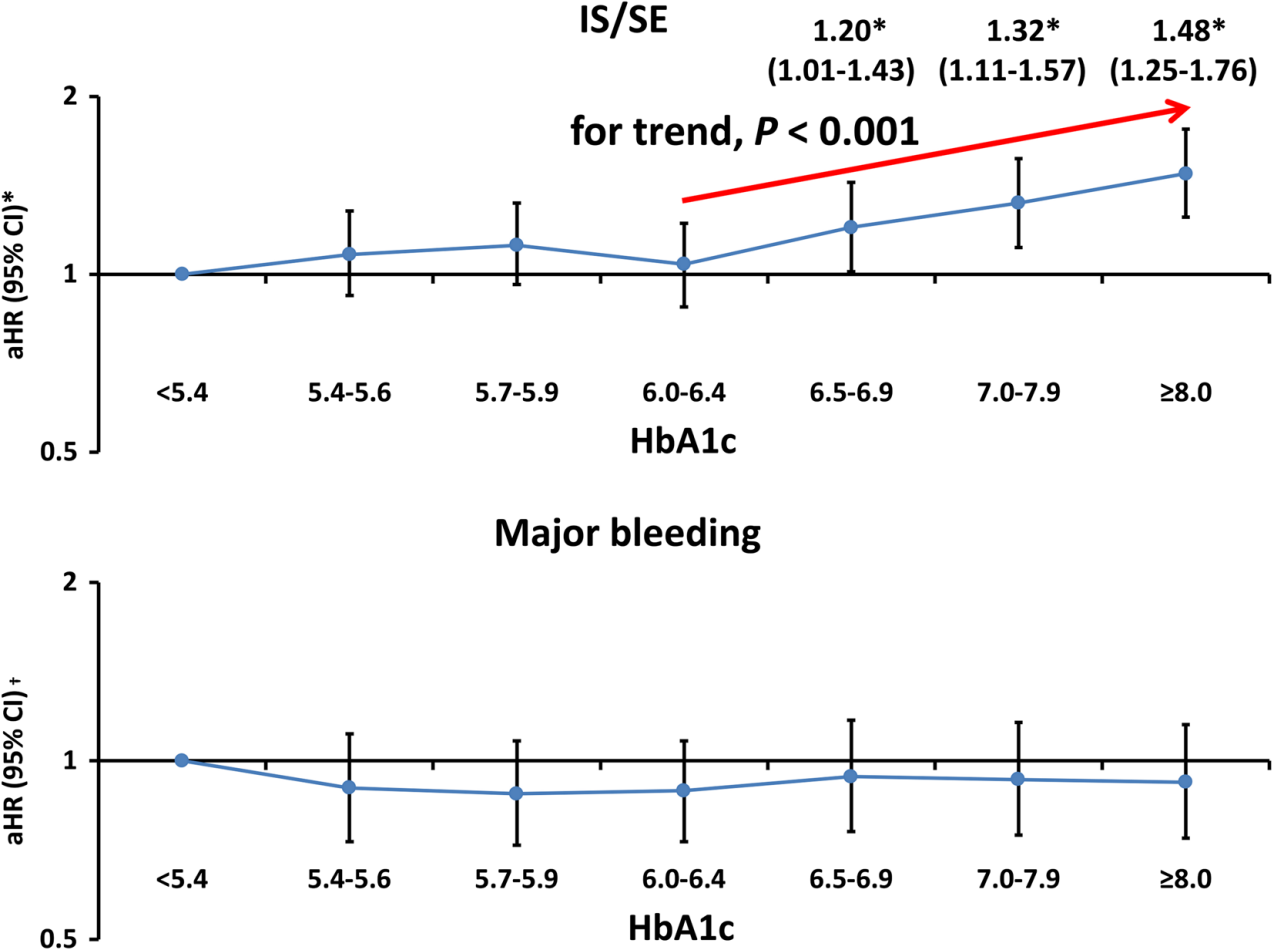
Risks of IS/SE and major bleeding for AF patients in different HbA1c categories. Compared with patients with an HbAc1 level of < 5.4%, the risk of IS/SE significantly increased when HbA1c level was higher than 6.5%, while the risk of IS/SE was similar for patients with HbA1c levels of 5.4%–5.6%, 5.7%–5.9%, and 6.0%–6.4%. In contrast to the risk of IS/SE, the risk of major bleeding was comparable across all HbA1c categories. *Risk of IS/SE was adjusted for CHA_2_DS_2_-VASc score, use of insulin or OHAs, use of warfarin or DOACs, and eGFR. **†**Risk of major bleeding was adjusted for HAS-BLED score, use of insulin or OHAs, use of warfarin or DOACs, and eGFR. AF = atrial fibrillation; CI = confidence interval; DOAC = direct oral anticoagulant; eGFR = estimated glomerular filtration rate; HbA1c = glycated hemoglobin A1c; aHR = adjusted hazard ratio; IS/SE = ischemic stroke or systemic embolism; OHA = oral hypoglycemic agent

Compared with patients with an HbA1c level of < 5.4%, the risk of IS/SE significantly increased when the HbA1c level was higher than 6.5% [adjusted hazard ratio (aHR) 1.20, 95% confidence interval (CI) 1.00–1.43, *P* = 0.0450 for HbA1c level of 6.5%–6.9%; 1.32, 95% CI 1.11–1.57, *P* = 0.0019 for HbA1c level of 7.0%–7.9%; and 1.48, 95% CI 1.25–1.76, *P* < 0.0001 for HbA1c level of $\ge$ 8.0%] after adjustments for CHA_2_DS_2_-VASc score, use of OACs, use of insulin/OHAs, and eGFR (Table 2 and Fig. 3). The risk of IS/SE was similar for patients with an HbA1c level of 5.4%–5.6%, 5.7%–5.9%, and 6.0%–6.4% compared with those with HbA1c < 5.4%. In contrast to the risk of IS/SE, the risk of major bleeding was comparable across all HbA1c categories (Table 2 and Fig. 3).

We also performed a sensitivity analysis in which all patients were categorized into three groups according to the ADA definitions by HbA1c levels, that is, non-DM (HbA1c < 5.7%), pre-DM (HbA1c 5.7%–6.4%), and DM groups (HbA1c $\ge$ 6.5%). The results are consistent with the main analysis, which indicated that the DM group [aHR: 1.26 (95% CI 1.13–1.41), *P* < 0.0001], but not the pre-DM group [aHR: 1.02, (95% CI 0.92–1.14), *P* = 0.6560], was associated with a significantly higher risk of IS/SE when compared with the non-DM group. In contrast, the risk of major bleeding did not differ significantly across these three groups (Table 3).

**Table 3:** Risks of hospitalized IS/SE and major bleeding among AF patients with non-diabetes, pre-diabetes, and diabetes

	Event rate/100 person-years (95% CI)	Crude		Adjusted	
		HR (95% CI)	*P* value	HR (95% CI)^a^	*P* value
IS/SE					
Non-diabetes (HbA1c < 5.7%)	1.28 (1.18–1.38)	1.00 (Reference)	–	1.00 (Reference)	–
Pre-diabetes (HbA1c 5.7–6.4%)	1.36 (1.27–1.44)	1.02 (0.92–1.13)	0.6968	1.02 (0.92–1.14)	0.6560
Diabetes (HbA1c ≥ 6.5%)	1.52 (1.43–1.61)	*1.26 (1.14–1.39)*	< 0.**0001**	*1.26 (1.13–1.41)*	< 0.**0001**

	Event rate/100 person–years (95% CI)	Crude		Adjusted	
		HR (95% CI)	*P* value	HR (95% CI)^b^	*P* value
Major bleeding					
Non-diabetes (HbA1c < 5.7%)	0.79 (0.71–0.87)	1.00 (Reference)	–	1.00 (Reference)	–
Pre-diabetes (HbA1c 5.7–6.4%)	0.76 (0.70–0.83)	0.94 (0.82–1.07)	0.3298	0.94 (0.83–1.08)	0.3881
Diabetes (HbA1c ≥ 6.5%)	0.82 (0.75–0.88)	1.11 (0.97–1.26)	0.1184	0.99 (0.86–1.15)	0.9122

^a^ Adjustments for CHA_2_DS_2_-VASc score, use of insulin or OHAs, use of warfarin or DOACs, and eGFR

^b^Adjustments for HAS-BLED score, use of insulin or OHAs, use of warfarin or DOACs, and eGFR

*AF* atrial fibrillation, *CI* confidence interval, *DOACs* direct oral anticoagulants, *eGFR* estimated glomerular filtration rate, *HbA1c* glycated hemoglobin A1c, *HR* hazard ratio, *IS/SE* ischemic stroke or systemic embolism, *OHAs* oral hypoglycemic agents

### Associations between HbA1c and risks of IS/SE and major bleeding in AF patients known to have DM or with unknown DM status

**Fig. 4:**
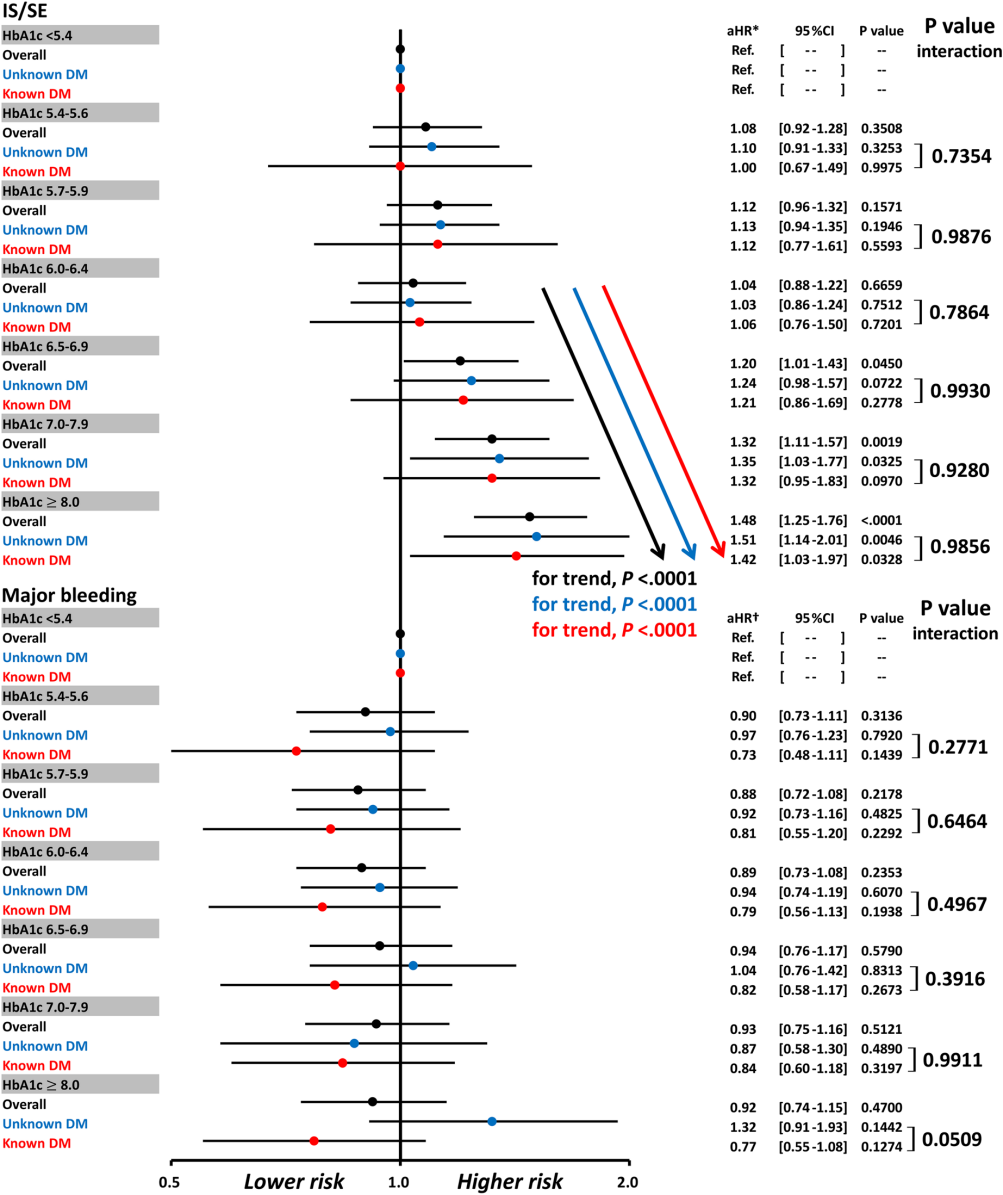
Risks of IS/SE and major bleeding in AF patients known to have DM or with unknown DM status across different HbA1c categories. There was no interaction observed with known DM or unknown DM status regarding the risk of IS/SE compared with patients with an HbA1c of < 5.4% in each HbA1c category (*P* interaction > 0.05 in all categories). There was also a significant trend for an increased risk of IS/SE among patients having HbA1c of 6.5%–6.9%, 7.0%–7.9%, and $\ge$ 8.0% for patients known to have DM or with unknown DM status (for trends, both *P* < 0.0001). There was no significant difference in the risk of major bleeding across all HbA1c categories in AF patients known to have DM or with unknown DM status. *Risk of IS/SE was adjusted for CHA_2_DS_2_-VASc score, use of warfarin or DOACs, and eGFR. **†**Risk of major bleeding was adjusted for HAS-BLED score, use of warfarin or DOACs, and eGFR. *AF* atrial fibrillation, *aHR* adjusted hazard ratio, *CI* confidence interval, *DM* diabetes mellitus, *eGFR* estimated glomerular filtration rate, *HbA1c* glycated hemoglobin A1c; *IS/SE* ischemic stroke/systemic embolism

We also performed analyses for AF patients known to have DM or with unknown DM status in each HbA1c category. Patients were defined as having “known DM” if DM had already been diagnosed before the HbA1c data were available (n = 14,097). The definition of “unknown” DM included patients without DM (HbA1c < 6.5%) and those with DM newly diagnosed (HbA1c $\ge$ 6.5%) (n = 19,939) after HbA1c data were available. There was no interaction observed for known or unknown DM regarding the risk of IS/SE compared with that in patients with an HbA1c of < 5.4% in each HbA1c category (*P* interaction > 0.05 in all categories) (Fig. 4). There was also a significant trend for an increased risk of IS/SE among patients having an HbA1c of 6.5%–6.9%, 7.0%–7.9%, and $\ge$ 8.0% for patients known to have DM or with an unknown DM status (for trends, both *P* < 0.0001) (Fig. 4). There was no significant difference in the risk of major bleeding across all HbA1c categories in AF patients known to have DM or with unknown DM status (Fig. 4).

### Associations between HbA1c and risk of IS/SE in AF patients treated with or without OACs

**Fig. 5:**
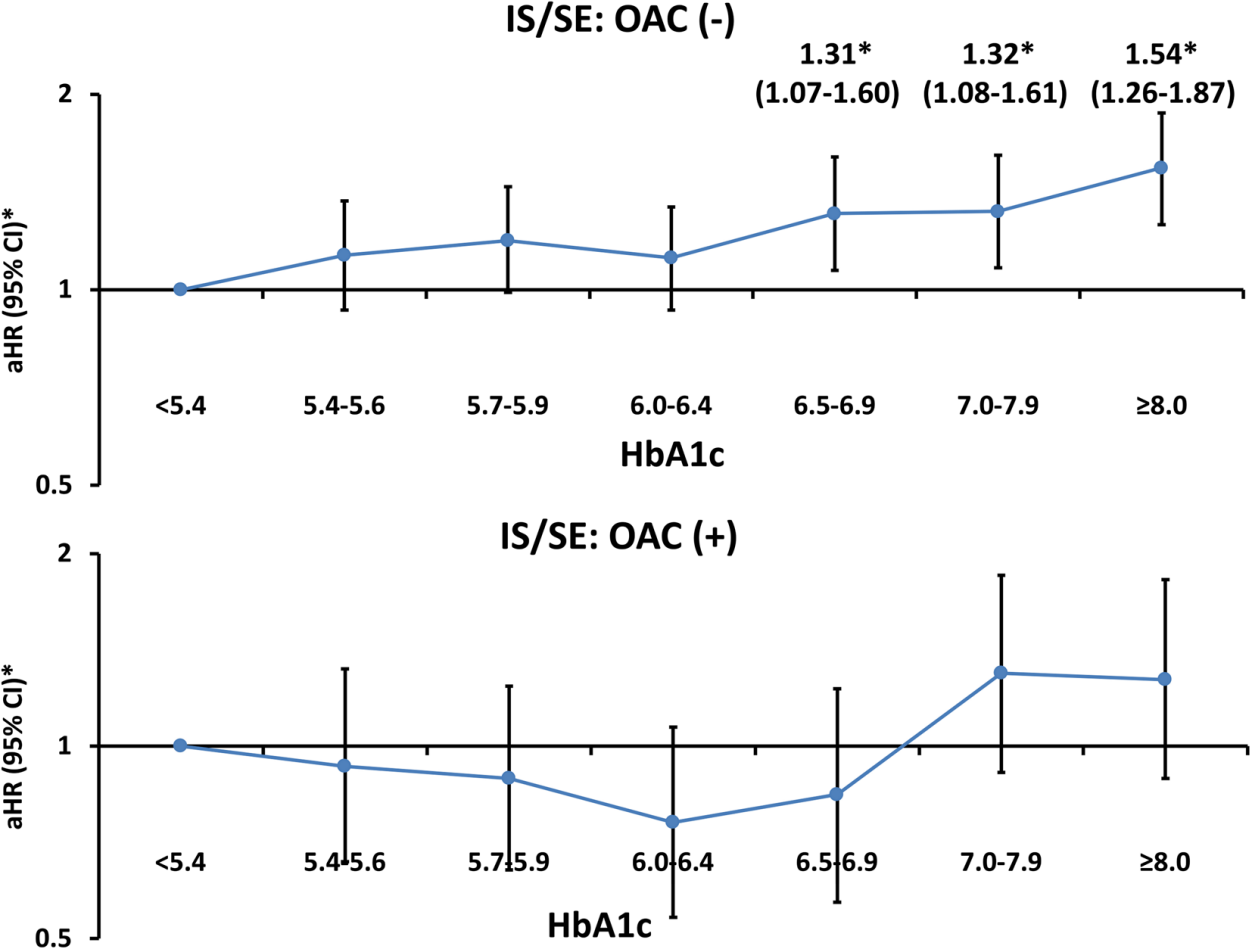
Risk of IS/SE for AF patients with/without taking OACs across different HbA1c categories. Results about the analysis of risk of IS/SE were generally consistent with the main analysis when we focused on AF patients not taking OACs (n = 24,931). However, for 9105 patients receiving OACs (3966 with DOACs and 5139 with warfarin), the risk of IS/SE was not higher among patients with a higher HbA1c level than in those with HbA1c of < 5.4%. *Risk of IS/SE was adjusted for CHA_2_DS_2_-VASc score, use of insulin or OHAs, and eGFR. *AF* atrial fibrillation, *aHR* adjusted hazard ratio, *CI* confidence interval, *DOAC* direct oral anticoagulant, *eGFR* estimated glomerular filtration rate, *HbA1c* glycated hemoglobin A1c, *IS/SE* ischemic stroke/systemic embolism, *OAC* oral anticoagulant

The results from analyzing the risk of IS/SE were generally consistent with the main analysis when we focused on AF patients not taking OACs (n = 24,931): aHR 1.31 (95% CI 1.07–1.60) (*P* = 0.0086) for HbA1c level of 6.5%–6.9%; 1.32 (95% CI 1.08–1.61) (*P* = 0.0076) for HbA1c level of 7.0%–7.9%; and 1.54 (95% CI 1.26–1.87) (*P* < 0.0001) for HbA1c level $\ge$ 8.0% compared with patients with HbA1c < 5.4% (Fig. 5). However, for 9105 patients receiving OACs (3966 with DOACs and 5139 with warfarin), the risk of IS/SE was not higher among patients with a higher HbA1c level than in those with HbA1c < 5.4% (Fig. 5).

### DOACs versus warfarin in AF patients with different HbA1c categories

**Fig. 6:**
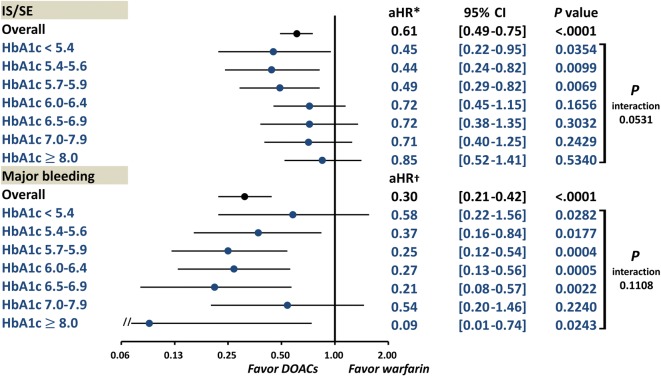
DOACs versus warfarin in AF patients with different HbA1c categories. Overall, DOACs were associated with a lower risk of IS/SE (adjusted HR 0.61, 95% CI 0.49–0.75, *P* < 0.0001) and major bleeding (adjusted HR 0.30, 95% CI 0.21–0.42, *P* < 0.0001) without interactions across different HbA1c categories (*P* interaction > 0.05 for both endpoints). *Risk of IS/SE was adjusted for CHA_2_DS_2_-VASc score, use of insulin or OHAs, and eGFR. ^**†**^Risk of major bleeding was adjusted for HAS-BLED score, use of insulin or OHAs, and eGFR. *AF* atrial fibrillation, *aHR* adjusted hazard ratio, *CI* confidence interval, *DOAC* direct oral anticoagulant, *eGFR* estimated glomerular filtration rate, *HbA1c* glycated hemoglobin A1c, *IS/SE* ischemic stroke/systemic embolism

Among the DOAC users, 727 (18.3%), 1176 (29.7%), 389 (9.8%), and 1674 (42.2%) patients were treated with apixaban, dabigatran, edoxaban, and rivaroxaban, respectively. Overall, DOACs were associated with lower risks of IS/SE (aHR 0.61, 95% CI 0.49–0.75, *P* < 0.0001) and major bleeding (aHR 0.30, 95% CI 0.21–0.42, *P* < 0.0001) without interactions across different HbA1c categories (*P* interaction > 0.05 for both endpoints) (Fig. 6).

## Discussion

### Main findings

In our study, we investigated the impacts of HbA1c level, in 34,036 AF patients with different glycemic control, on the risks of IS/SE and major bleeding. The main findings of this study are as follows: (i) The HbA1c level, irrespective of the presence of established DM or use of insulin/OHAs, is associated with a significantly increased risk of IS/SE once the HbA1c level is $\ge$ 6.5% in AF patients. (ii) The risk of major bleeding is comparable across all HbA1c categories. (iii) The associations between HbA1c levels and the risk of IS/SE among AF patients seemed to be attenuated with OAC use. (iv) Compared with warfarin, DOACs are associated with lower risks of IS/SE and major bleeding in each HbA1c category.

### Associations between HbA1c levels and risks of IS/SE and major bleeding

Several studies investigated the associations between HbA1c levels and risk of systemic thromboembolism in AF patients [10–12]. Saliba et al. found that the HbA1c level was associated with a higher risk of ischemic stroke in a dose–response manner among 11,176 AF patients with type 2 DM, which is consistent with our findings [10]. However, the cut-off level of HbA1c at which the risk of thromboembolism significantly increased was 7%, rather than 6.5% as demonstrated in our study. Fangel et al. studied 5386 AF patients with type 2 DM and showed that a higher risk of thromboembolism was observed among patients with an HbA1c level of > 6.6% compared with those with an HbA1c level of ≤ 6.5% [12]. However, this association was only significant among patients with a DM duration of < 10 years. Ashburner et al. studied 2101 AF patients with DM in the ATRIA California community-based cohort and showed that a DM duration of > 3 years was a more important predictor of ischemic stroke than HbA1c level itself [11]. In our study, a higher risk of IS/SE was observed for Asian AF patients having an HbA1c level of > 6.5%. However, whether more aggressive glycemic control to achieve an HbA1c level of < 6.5%, rather than < 7.0% suggested by the American Diabetes Association for the general diabetic population [22], could lower the risk of IS/SE in AF patients deserves further study. Moreover, we also demonstrated that the risk of major bleeding did not differ significantly in different HbA1c categories, and these findings support the commonly used bleeding prediction scheme, HAS-BLED score, which does not include DM as a risk factor component for bleeding [13]. However, the meta-analysis of four pivotal DOAC trials showed that the rates of major bleeding were 6.35% in patients with DM vs. 5.40% in those without DM (*P* < 0.0001) [23]. A high prevalence of comorbid hypertension and renal dysfunction due to advanced diabetes, which are indeed included as risk factor components of the HAS-BLED score, may result in a higher risk of bleeding for DM patients. We should pay attention to the overlap of these factors with DM when managing anticoagulated diabetic AF patients.

Furthermore, we found a high rate of prescription of nonsteroidal anti-inflammatory drugs (NSAIDs) in our AF population across different HbA1c categories (Table 1). A previous study also showed that NSAIDs were commonly used among AF patients who initiated OACs [24]. Although most AF patients took NSAIDs transiently rather than regularly in the present study, previous studies already indicated that even transient use of NSAIDs could pose a serious risk of both cardiovascular events and bleeding [25, 26]. Kent et al. studied the effects of NSAIDs in the RE-LY trial, which included 18,113 patients, 2279 of whom had used NSAIDs at least once during the follow-up. The risks of stroke/SE [HR: 1.50 (95% CI 1.12–2.01), *P* = 0.007], major bleeding [HR: 1.68 (95% CI 1.40–2.02), *P* < 0.0001], and hospitalization [HR: 1.64 (95% CI 1.51–1.77), *P* < 0.0001] were significantly higher with NSAIDs, irrespective of receiving DOACs or warfarin [27]. Further prospective study is necessary to investigate this issue among the diabetic AF population.

### Associations between HbA1c levels and risk of IS/SE among patients on OACs

Interestingly, our study also showed that the use of OACs may attenuate the dose–response linear association between HbA1c and the risk of IS/SE in AF patients. Previous randomized studies, ADVANCE and ACCORD trials [28, 29], did not show benefits of intensive glycemic control on reducing major macrovascular events; one explanation of this is that the widespread use of statins may play a more important role in stroke prevention for diabetic patients. Similarly, a possible explanation of our finding is that the most important and effective method to reduce AF-associated stroke is the use of OACs; therefore, the impact of glycemic control on the risk of IS/SE was attenuated. This is partly supported by the post hoc analysis of the ROCKET-AF trial, indicating that the presence of DM was associated with a higher risk of myocardial infarction (2.60% vs. 1.57%), but not a higher risk of IS/SE (2.99% vs. 3.52%), among AF patients fully anticoagulated with rivaroxaban or warfarin [30]. However, our findings should not be misinterpreted as showing that glycemic control is not important for anticoagulated AF patients. The key message is that OACs should be prescribed for AF patients whenever indicated, along with efforts toward glycemic control.

### Comparisons of DOACs and warfarin in different HbA1c categories

We also demonstrated that DOACs were associated with lower risks of IS/SE and major bleeding than warfarin across different HbA1c categories. The RE-LY and ARISTOTLE studies indicated that dabigatran at 110 mg twice daily and apixaban at 5/2.5 mg twice daily were associated with significantly lower risks of major bleeding than warfarin [31, 32]. However, the benefits of reduced major bleeding for dabigatran at 110 mg or apixaban at 5/2.5 mg over warfarin were not significant in AF patients with DM in the post hoc analysis of these pivotal trials [15, 33]. Actually, there were no clear data showing the benefits of DOACs over warfarin regarding the risk of major bleeding in AF patients with DM [34, 35]. As we know, warfarin may have several drawbacks, especially in diabetic AF patients. Variability of glycemic level in diabetic patients may affect the pharmacokinetics of warfarin, which may increase the risk of INR values outside the target range in diabetic AF patients [36]. Indeed, the presence of diabetes was independently associated with poor TTR in warfarin-treated AF patients [37]. Furthermore, warfarin inhibits the vitamin K-dependent gamma-glutamyl carboxylation of proteins, including matrix Gla protein and osteocalcin (bone Gla protein); therefore, the use of warfarin may increase the risk of vascular calcification and osteoporotic bone fracture in diabetic AF patients [38]. Our study supported the advantages of DOACs over warfarin regarding efficacy and safety in AF patients across a broad spectrum of HbA1c levels [34, 38, 39]. However, further randomized and prospective studies assessing the safety of DOACs vs. warfarin in patients with concomitant AF and type 2 DM are warranted.

### Study limitations

There were several limitations in our study. First, this was a retrospective study and 36,372 AF patients whose HbA1c data were lacking were excluded from the analysis. Therefore, some biases may have been present and whether patients whose HbA1c data were available could represent the whole AF population remained unclear. Furthermore, the clinical characteristics of the patients were different across the different HbA1c categories. Although we adjusted for CHA_2_DS_2_-VASc or HAS-BLED score, use of insulin or OHAs, use of warfarin or DOACs and eGFR in the multivariate Cox regression models, other residual confounding factors were probably present. Second, the CGMH database lacks some patient information, including physical activity, alcohol intake, smoking, and blood pressure. Therefore, it is unclear whether the impacts of HbA1c levels on the risk of IS/SE were partially confounded by these factors. Besides, since data about echocardiography were lacking in around 40% of patients, we were not able to further characterize patients with heart failure as having heart failure with reduced or preserved ejection fraction. Third, we showed a higher risk of IS/SE for patients with a higher HbA1c level, but whether aggressive glycemic control to achieve an HbA1c level of < 6.5% can reduce the risk of AF-associated stroke deserves further study. Fourth, since DM is a risk factor of atherosclerosis, a considerable proportion of ischemic strokes occurring among AF patients with diabetes could be due to lacunar and atherothrombotic cerebral infarction rather than cardiogenic thromboembolism. However, information about the etiologies of ischemic stroke was not available in our database. Fifth, a high percentage (73%) of AF patients did not receive long-term OACs for stroke prevention in our AF cohort. The mean CHA_2_DS_2_-VASc score of these patients was 3.2 (standard deviation 1.9) and ~ 80% of them had a CHA_2_DS_2_-VASc score ≥ 2. Actually, the underuse of OACs is a global issue, especially for Asian AF patients [40, 41]. Multiple factors, such as the fear of bleeding and the inconvenience of warfarin, are related to the underuse of OACs. However, the exact causes of why those patients at risk of stroke did not receive OACs in our study were unclear. Lastly, data on TTR of INR for AF patients treated with warfarin were not available in our analysis. In general, Asians taking warfarin have difficulty in maintaining INR in the target range of 2–3, and their TTR is substantially lower than that of patients in other parts of the world [41]. In addition, persistence of OAC treatment was assumed after the first prescription of an OAC. Moreover, prescriptions of other medications were confined to those taken at least once within 3 months before the index date, and the compliance was not verified. Therefore, residual confounding factors including the quality and persistence of OACs and other medications across the exposure groups could not be confirmed.

## Conclusions

For AF patients, the risk of IS/SE significantly increased once the HbA1c levels exceeded 6.5%, while the risk of major bleeding was comparable across all HbA1c levels. The use of OACs may attenuate the associations between HbA1c levels and the risk of IS/SE. Compared with warfarin, DOACs were more effective and safer across broad HbA1c categories. In addition to prescriptions of DOACs when indicated, more aggressive glycemic control to achieve an HbA1c level of < 6.5% may be considered for eligible AF patients, which should be tested in further prospective studies.

## Data Availability

The datasets used and/or analyzed during the current study are available from the corresponding author on reasonable request.

## Abbreviations

ACEI: angiotensin-converting enzyme inhibitor
AF: Atrial fibrillation
ALT: Alanine aminotransferase
APT: Antiplatelet agent
ARB: Angiotensin II receptor antagonist
CHA_2_DS_2_-VASc: Congestive heart failure, hypertension, age 75 years or older, diabetes mellitus, previous stroke/transient ischemic attack, vascular disease, age 65 to 74 years, female
DOAC: Direct oral anticoagulant
eGFR: Estimated glomerular filtration rate
HAS-BLED: Hypertension, abnormal renal or liver function, stroke, bleeding history, labile INR, age 65 years or older, and antiplatelet drug or alcohol use
HbA1c: Glycated hemoglobin A1c
OHA: Oral hypoglycemic agent
NSAID: Nonsteroidal anti-inflammatory drug
TIA: Transient ischemic attack
DM: Type 2 diabetes mellitus
